# Double‐strand break repair based on short‐homology regions is suppressed under terminal deoxynucleotidyltransferase expression, as revealed by a novel vector system for analysing DNA repair by nonhomologous end joining

**DOI:** 10.1002/2211-5463.12001

**Published:** 2016-01-04

**Authors:** So Maezawa, Saori Nakano, Takaaki Kuniya, Osamu Koiwai, Kotaro Koiwai

**Affiliations:** ^1^ Department of Applied Biological ScienceFaculty of Science and TechnologyTokyo University of ScienceNodaChibaJapan

**Keywords:** DNA double‐strand break, DNA polymerase λ, DNA polymerase μ, DNA polymerase X, nonhomologous end joining, terminal deoxynucleotidyltransferase

## Abstract

We have constructed a novel, nonhomologous end‐joining (NHEJ) assay vector (NAV), containing *mKate2*,* Venus* and *ccdB* genes. Cotransfection of NAV with a construct expressing the restriction enzyme I‐*Sce*I generated a double‐strand break (DSB) in NAV that excised *mKate2* and *ccdB*. Repair of this DSB produced an intact vector that expressed Venus, a green fluorescent protein. Because cells bearing the repaired NAV lacked the *ccdB* gene which slows cell proliferation, the cultures were enriched in cells containing repaired DSBs. DNA sequence analysis of the DSB junctions indicated that the repair was carried out mainly by using the closest homology sequence. Use of the NAV yielded rapid results within 3 days after transfection. We then used the NAV to analyse NHEJ in cells overexpressing terminal deoxynucleotidyltransferase (TdT). The results indicated that TdT suppresses DNA repair that is based on short (one‐ or two‐base) homology regions, to efficiently add deoxynucleotides during VDJ recombination in lymphoid cells.

**Abbreviations**
C‐NHEJ: classical nonhomologous end joining
DSB: DNA double‐strand break
*E. coli*: *Escherichia coli*
FBS: foetal bovine serum
Lig IV: DNA ligase IV
MEM: minimum essential medium
NAV: nonhomologous end‐joining assay vector
NHEJ: nonhomologous end joining
NT: nucleodidyltransferase
PCNA: proliferating cell nuclear antigen
PCR: polymerase chain reaction
pol λ: DNA polymerase λ
pol μ: DNA polymerase μ
TdIF1: TdT interacting factor 1
TdT: terminal deoxynucleotidyltransferase

A DNA double‐strand break (DSB), if unrepaired or misrepaired, can ultimately lead to cell death or cancer. Classical nonhomologous end joining (C‐NHEJ) is a cell‐cycle‐independent mechanism that repairs DSBs by joining two broken ends of the DNA 1, 2, 3, 4. C‐NHEJ involves Ku70/Ku80, DNA‐PKcs, Artemis, DNA ligase IV (Lig IV), XRCC, XLF and the DNA polymerases μ (pol μ) and λ (pol λ) 1, 2, 3, 4. The DNA ends are resected by the Artemis nuclease, the gap between the two DNA ends is filled by pol μ/λ 5, and then the ends are joined by Lig IV, XRCC and XLF.

There are two ways to detect NHEJ in cells. One is to use a plasmid that induces a DSB and its repair by NHEJ 6, and the other is to introduce into a chromosome a specific DSB site that can be used as an NHEJ reporter 7, 8. Here, using the first strategy, we constructed a plasmid‐based NHEJ assay vector (NAV) that served as a substrate for DSB repair. The restriction enzyme I*‐Sce*I induced a DSB at a specific DNA sequence in the NAV. Cells expressing the cut and repaired NAV expressed Venus and fluoresced green, while cells bearing the original, intact NAV expressed mKate2 and fluoresced red. To enrich for *Escherichia coli* cells containing repaired NAVs, we also inserted a *ccdB* gene into the NAV construct between the *mKate2* gene and the poly‐A sequence of the vector, so that I‐*Sce*I digestion removed the *ccdB* gene from the NAV along with the *mKate2* gene. The *ccdB* gene product binds to DNA gyrase, inducing illegitimate recombination and decreasing cell proliferation 9, so any cells bearing uncleaved NAV would proliferate more slowly than those with repaired DSBs. We then used the NAV to identify DNA sequences at the repaired junctions of DSBs produced by I*‐Sce*I. Our results indicated that the NAV system is a powerful tool for the rapid analysis of NHEJ.

During VDJ recombination in lymphoid cells, terminal deoxynucleotidyltransferase (TdT) randomly adds deoxynucleotides between the V and D or D and J DNA segments of the immunoglobulin (Ig) or T‐cell receptor genes to synthesize the N region, after which the DSBs are repaired by C‐NHEJ 10, 11. We used the NAV system to analyse the C‐NHEJ repairs by pol μ/λ when TdT was overexpressed in mammalian cells, and found that NHEJ using short‐homology regions of one or two bases was suppressed.

## Materials and methods

### Plasmid construction

The pEB vector and I‐*Sce*I expression plasmid were given by Y. Miwa (Tsukuba University). The *mKate2* and *Venus* genes were subcloned into the pEB vector. A *ccdB* gene cloned from the *E. coli* F plasmid (purchased from Life Technologies Japan, Tokyo, Japan) was amplified by PCR using a forward primer containing a *Bgl* II site and a reverse primer containing an *Eco*R I site, and the amplified gene was inserted between the *Bgl* II and *Eco*R I sites of the pEB vector.

### Cell culture

U2OS human osteosarcoma cells were cultured in Alpha Modified Eagle's Minimum Essential Medium (αMEM) supplemented with 10% foetal bovine serum (FBS) at 37 °C and 5% CO_2_ in a humidified incubator.

### Transfection

U2OS cells (1 × 10^6^ cells) were cotransfected with 3 μg of NAV and 1 μg of I‐*Sce*I expression vector in a 60‐mm dish using the X‐tremeGENE HP DNA Transfection Reagent (Roche, Basel, Switzerland), according to the manufacturer's instructions. To overexpress TdT, 1 μg of FLAG‐TdT‐expression vector or control vector without the TdT gene were cotransfected together with NAV and the I‐*Sce*I vector. The overexpressed FLAG‐TdT was analysed by immunoblotting using a mouse monoclonal anti‐TdT antibody. About 10 ng of TdT was expressed in 1 × 10^6^ cells. The transfection efficiency was normalized to the coexpressed mKate2 using a rabbit monoclonal anti‐mKate2 antibody. The transfected cells were incubated for 36 h and then analysed by fluorescence microscopy, flow cytometry, or NAV isolation. Live cells were directly observed in the dish using the LCV110 bio‐imaging system (Olympus, Tokyo, Japan).

### Flow cytometric analysis

U2OS cells were treated with trypsin and suspended in phosphate‐buffered saline (PBS) containing 1% FBS and 0.05% NaN_3._ Cells emitting Venus and/or mKate2 fluorescence were counted on a FACSCanto II (Becton Dickinson and Company, New Jersey, USA) using a 488‐nm argon laser with a 530/30 bandpass (BP) filter or a 633‐nm helium‐neon laser with a 660/20 BP filter respectively. Data were analysed using flowjo software (FlowJo, Oregon, USA).

### Breakpoint analysis

Repaired NAVs were isolated from U2OS cells and transferred into *E. coli* DH5α cells. The repaired DNA regions in the NAV were amplified by colony‐directed PCR. The PCR products were purified by ExoSAP 6. Briefly, a 2‐μl reaction mixture containing 1 μg of PCR products was mixed with 1 μL of ExoSAP reagent containing 0.005 U of exonuclease I (NEB) and 0.0025 U of Shrimp Alkaline Phosphatase (Roche). DNA sequences were determined by the Sanger method with the reverse primer 5′‐TTCAGGGTCAGCTTGCCGTA‐3′, on an ABI 3100 capillary DNA sequencer (Life Technologies Japan).

## Results

### NAV construction

**Figure 1:**
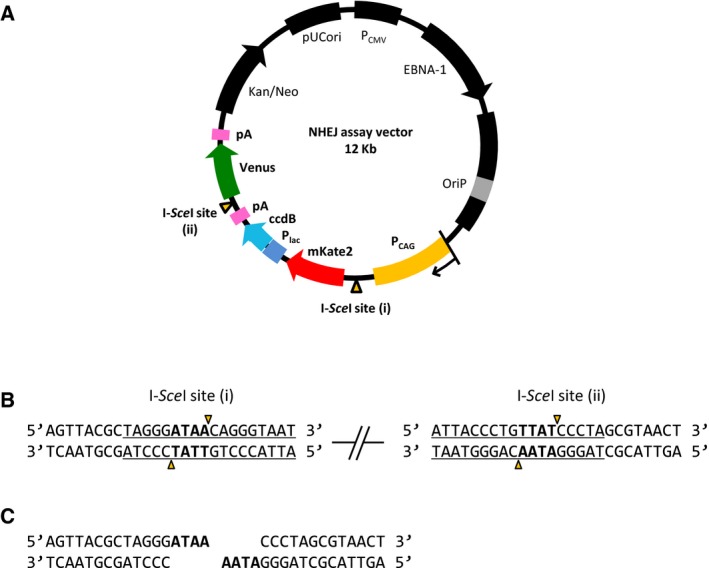
The NHEJ assay vector (NAV). (A) Orange triangles indicate I‐*Sce*I recognition sites. *P_CAG_* (yellow): CAG promoter; *P_lac_* (blue): lactose promoter; pA (pink): poly‐A signal. For the assay, U2OS cells were cotransfected with the NHEJ assay vector (NAV) and an I‐*Sce*I expression vector. (B) I‐*Sce*I recognized 18 base pairs (underlined) to produce a 3′ hydroxyl overhang of four bases (bold). (C) The DNA ends produced by I‐*Sce*I digestion.

In this study, the fluorescent proteins Kate2 and Venus, which fluoresce red and green, respectively, were used to distinguish between the original (intact) and successfully repaired NAVs. Expressing the restriction enzyme I‐*Sce*I generated DSBs and excised the *mKate2* gene. As illustrated in Fig. 1A, *mKate2* transcription was controlled by the *CAG* promoter (*P*_*CAG*_). I‐*Sce*I excised *mKate2*, preventing mKate2 expression and causing cells to lose their red fluorescence. Sequences encoding the two I‐*Sce*I recognition sites were inserted in reverse orientation (Fig. 1B) so that both ends of the break generated by I‐*Sce*I digestion had identical protruding DNA sequences (5′‐ATAA) (Fig. 1C), to prevent base‐pair formation and ligation. The DNA ends could only be rejoined when they were properly processed and repaired by NHEJ in the cells. Successful repair of the vector placed the *Venus* gene downstream of *P*_*CAG*_, causing the cell to express Venus and fluoresce green. When multiple NAVs were introduced into a cell and both repaired and unrepaired vectors were present in the same cell, the red and green fluorescence merged and the cell fluoresced yellow.

To enrich for the repaired vectors, a *ccdB* gene under control of the *Lac* promoter (*P*_*lac*_) was inserted between the *mKate2* gene and the poly‐A signal in the vector. This gene was also excised by I‐*Sce*I. Therefore, only *E. coli* cells containing successfully repaired NAVs that no longer expressed the *ccdB* gene, could survive and proliferate. This vector system allowed us to evaluate the level of NHEJ activity in live cells.

### NAV analysis of NHEJ in U2OS cells

**Figure 2:**
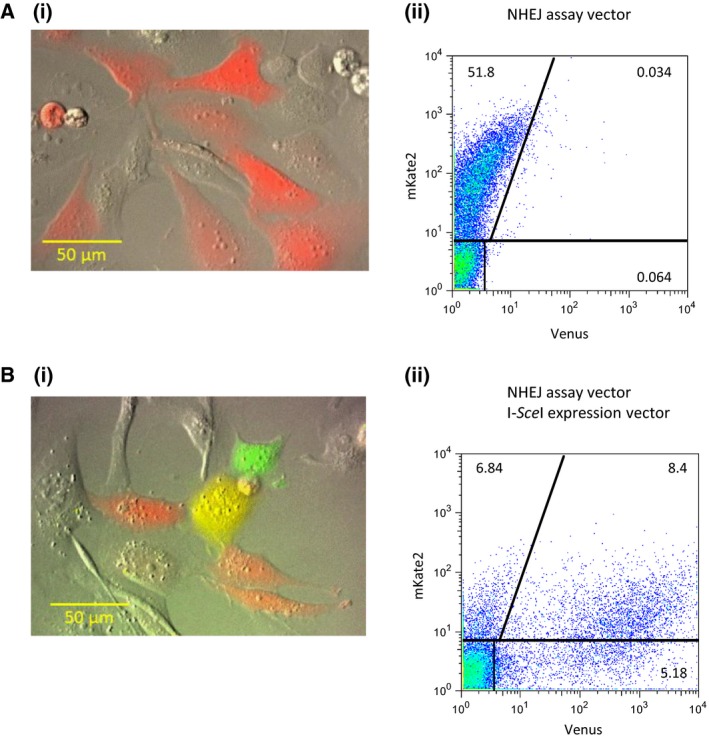
Analysis of mKate2 and Venus expression by fluorescence microscopy and flow cytometry. U2OS cells were transfected with NAV alone (A) or NAV and an I‐*Sce*I expression vector (B). Thirty‐six hours later, the cells were analysed by fluorescence microscopy (Ai and Bi) and flow cytometry (Aii and Bii).

U2OS cells transfected with the NAV vector uniformly fluoresced red, indicating mKate2 expression (Fig. 2A). When U2OS cells were cotransfected with NAV and an I‐*Sce*I expression vector under our experimental conditions, 34% of the transfected cells emitted red fluorescence, while 41% and 25% emitted yellow and green fluorescence respectively (Fig. 2B). We also observed that in cells emitting yellow fluorescence, the colour gradually changed to green. This may indicate that it takes time for I*‐Sce*I to completely digest all of the I*‐Sce*I recognition sites, that the process was limited by I*‐Sce*I expression in the cell, or both. It is also possible that the half‐life of mKate2, which is longer than that of Venus, contributed to the initial yellow fluorescence in cells containing repaired NAVs 7. That is, even when all of the recognition sites were digested, the cell would still fluoresce red until all of the mKate2 was completely degraded. Thus, the initial red fluorescence gradually changed to yellow, and finally to green (Fig. S1). FACS analysis revealed that the efficiency of repair was 47%, which was calculated as the ratio of the number of Venus‐expressing cells to mKate2‐ and Venus‐expressing cells.

**Figure 3:**
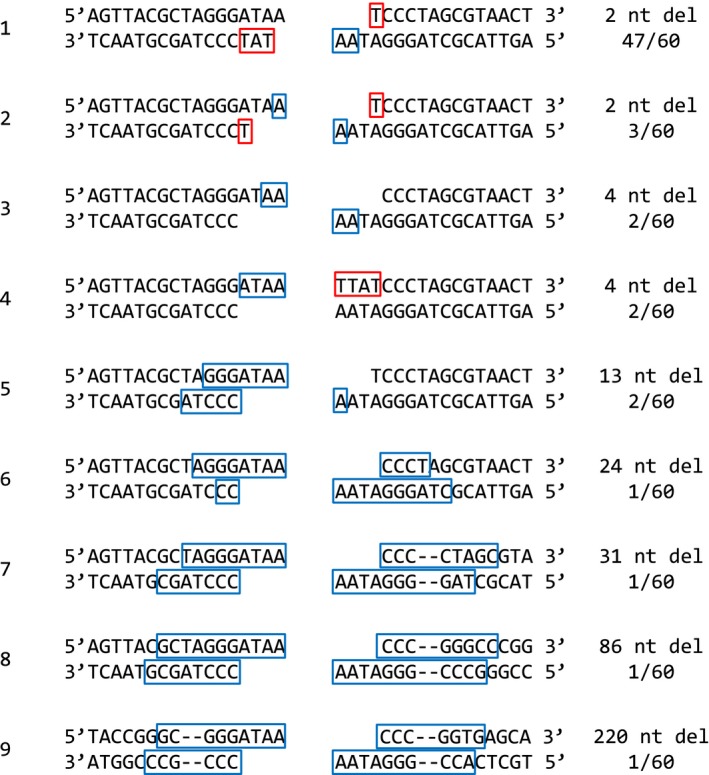
DNA sequences at the repaired DSB junctions. The DNA sequences at 60 DSB junctions repaired by NHEJ were determined. Excised nucleotides are outlined in blue, with the number of excised nucleotides shown at right (nt del). Red squares indicate nucleotides filled in by DNA polymerase. Ratios shown at right indicate how many instances of each sequence were found in a sample size of 60.

We then isolated the NAV vectors from the transfected U2OS cells, and transferred them into *E. coli* DH5α cells. We amplified the DSB junction regions by direct PCR, and determined the DNA sequences of the amplified junctions using the PCR products as templates. Of 60 DNA samples, 47 contained the sequence 5′‐ATAAT at the junction, suggesting that the 5′‐AA in one of the protruding ends was excised to form one A‐T base pair, and the single‐strand DNA regions were then filled in by pol μ/λ (Fig. 3). As shown in Fig. 3, eight other DNA sequences were also detected, but there were three or fewer of each. From these results, we concluded that the cells mainly repaired DSBs using short‐homology regions of one or two bases present at the DSB ends, and that pol μ/λ actively synthesized DNA to fill in the gaps.

We also noted that no base replacement occurred at the DNA ends *in vivo*, even in the absence of short homology (Fig. 3, example 4). Thus, the pol μ/λ activities accurately repaired DSBs by generating the correct base pairs *in vivo*.

As Ligase IV is used in the C‐NHEJ pathway, we confirmed that we were observing C‐NHEJ by knocking‐down Ligase IV. As shown in Table 1, in the Ligase IV‐knockdown cells, 46% (23 of 50 DNA samples) of the repaired plasmids had repaired the DSBs using short homology and 28% (14 of 50 samples) had deletions longer than 60 bases. These results strongly suggested that the DSBs in the NAV were mainly repaired through the C‐NHEJ pathway, but when C‐NHEJ was not available, another repair pathway (alt‐NHEJ) was used 12, 13.

**Table 1:**
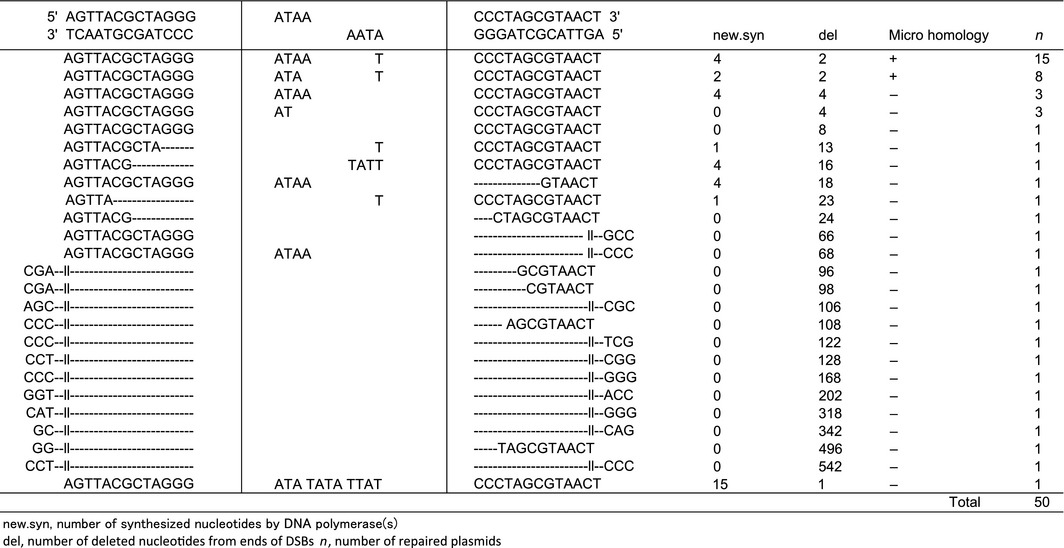
DNA sequences of the repaired DSB junctions when DNA Ligase IV is knocked down

### TdT suppression of short homology‐based NHEJ

We next used our NAV system to analyse the C‐NHEJ in TdT‐overexpressing cells. TdT's function results in a high divergence of DNA sequences at DSBs, unlike normal C‐NHEJ, which accurately conserves the original DNA sequences at DSB junctions. We therefore suspected that normal short‐homology‐based DNA repair might be suppressed under TdT expression. To test this theory, we cotransfected NAV and a TdT expression vector into U2OS cells and analysed the DNA sequences at the repaired DSBs. As shown in Table 2, 86% of the repaired plasmids contained extra nucleotides added by TdT. Therefore, as we had speculated, there was very little short homology‐based DNA repair in cells cotransfected with NAV and the TdT vector compared to cells transfected with NAV alone: short homology‐based DSB repair was found in 52 of 60 cases (87%) in cells transfected with NAV only, but in only 3 of 72 cases (0.4%) in cells cotransfected with the TdT expression vector. The frequency of C‐NHEJ in the TdT‐overexpressing cells was similar to that in control cells, as shown by real‐time PCR. Thus, our data strongly suggested that short homology‐based DSB repair is suppressed under TdT expression.

**Table 2:**
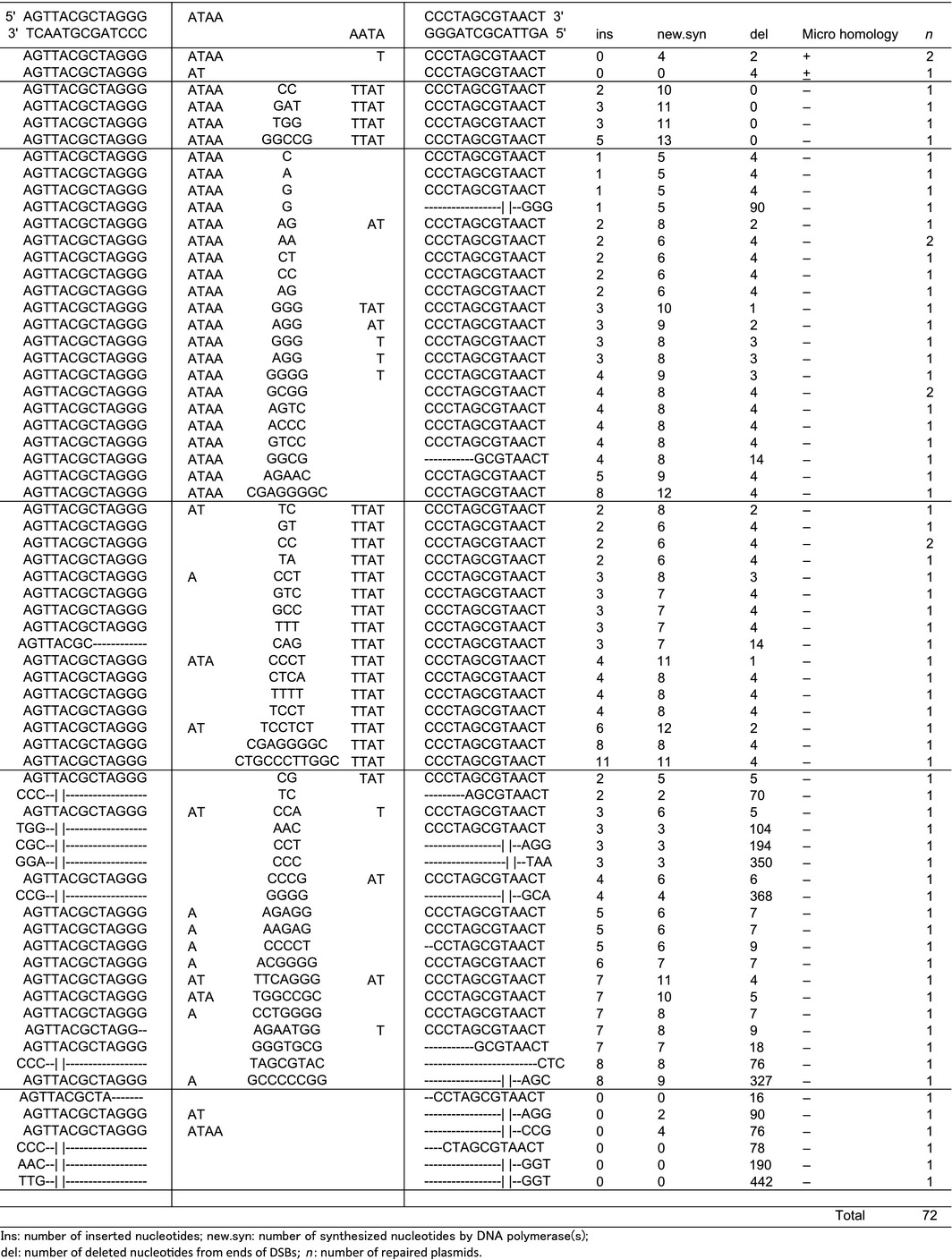
DNA sequences of the repaired DSB junctions when TdT is overexpressed

## Discussion

We have constructed a novel plasmid‐based NHEJ assay vector, NAV. Using conventional plasmids, it takes more than a month to analyse plasmid–based NHEJ, because it is difficult to obtain enough repaired plasmids for analysis. Here, we overcame this problem by introducing the *ccdB* gene, to enrich for the repaired vectors. The NAV system generates results very quickly, taking just 3 days from transfection to determination of the DNA sequence of the repaired DSBs. In addition, it was not necessary to perform PCR to obtain the DNA fragments containing DSBs, so we could skip the step of purifying amplified DNA fragments from the agarose gel after PCR, and directly introduced the plasmids from mammalian cells into *E. coli*. In our hands using conventional plasmids, the agarose‐gel‐purified amplified DNA fragments derived from the intact plasmids were always contaminated. Using the NAV system, such contamination was not a problem. Moreover, we could easily discriminate the repaired from the intact plasmids by the green Venus and red mKate2 proteins, respectively, and used these colours to follow the time course of repair in cells. Notably, our NAV plasmid‐based results correlated well with recently reported findings using a chromosome‐based DSB repair detection system 8. Thus, the NAV is especially useful for quickly and accurately analysing the DNA sequences at DSBs.

Our data indicated that when extensive deletions (13 bases or more) were generated in the double‐stranded DNA surrounding the DSB, DNA polymerase did not repair the DSB. Moreover, no additional nucleotides were inserted at the DSB junctions. Both pol μ and pol λ have distributive DNA polymerase activity, namely, nucleotidyltransferase (NT) activity, *in vitro* 14, 15. Therefore, our results suggest that the NT activities of pol μ/λ may not be used in most cell types *in vivo*; these polymerases may be specifically active in lymphocytes and suppressed in other cell types.

DSBs generated during VDJ recombination in lymphoid cells are repaired by C‐NHEJ, in which the DNA polymerases of pol μ, pol λ, or TdT function. Since pol λ mainly functions in the homology‐based DSB repair 16, 17 and DSBs are not repaired by the short homology‐based DNA repair system when TdT is overexpressed, TdT expression may inhibit the pol λ activity. In this situation, since DSBs are not repaired quickly by the pol λ homology activity, exonuclease(s) can delete several nucleotides from the DSB ends, and the percentage of plasmids with deletions should increase. In fact, in our TdT‐overexpressing cells, 23% of the repaired plasmids had deletions of more than 10 nucleotides, compared to 10% of the repaired plasmids in cells without TdT overexpression.

When we knocked down pol μ or pol λ, DSBs were still repaired with normal efficiency, indicating that these DNA polymerases act complementally in DSB repair. Pol μ is the only DNA polymerase that can repair the gaps without using homology at the DNA ends of DSBs 8, 18. Therefore, in TdT‐overexpressing cells, since the pol λ activity is suppressed, pol μ may synthesize the double‐stranded DNA 19, 20.

Our data strongly suggest that short homology‐based DSB repair was suppressed in TdT‐overexpressing cells. This suppression is reasonable, because it would promote diversity at the N regions when TdT is expressed in lymphoid cells. Thus, our findings showed that the NAV system is a powerful tool for analysing the functions of proteins involved in C‐NHEJ. Experiments to elucidate the roles of TdT interacting factor 1 (TdIF1), TdIF2 and PCNA 21, 22, 23 in C‐NHEJ by knocking them down in TdT‐overexpressing cells containing NAV, are presently underway in our laboratory.

## Author contributions

SM and OK conceived and designed the project; SN and TK acquired the data, SM, SN and TK analysed and interpreted the data, SM, OK and KK wrote the paper.

## Supporting information


**Fig. S1.** Cell fluorescence over time.

**Video S1.** Time‐lapse imaging of cell fluorescence.
