# Supplementary figures and images for: Double‐strand break repair based on short‐homology regions is suppressed under terminal deoxynucleotidyltransferase expression, as revealed by a novel vector system for analysing DNA repair by nonhomologous end joining
Source: FEBS Open Bio. 2016 Jan 4;6(1):16–23. doi: 10.1002/2211-5463.12001 (PMC4794791; doi:10.1002/2211-5463.12001)

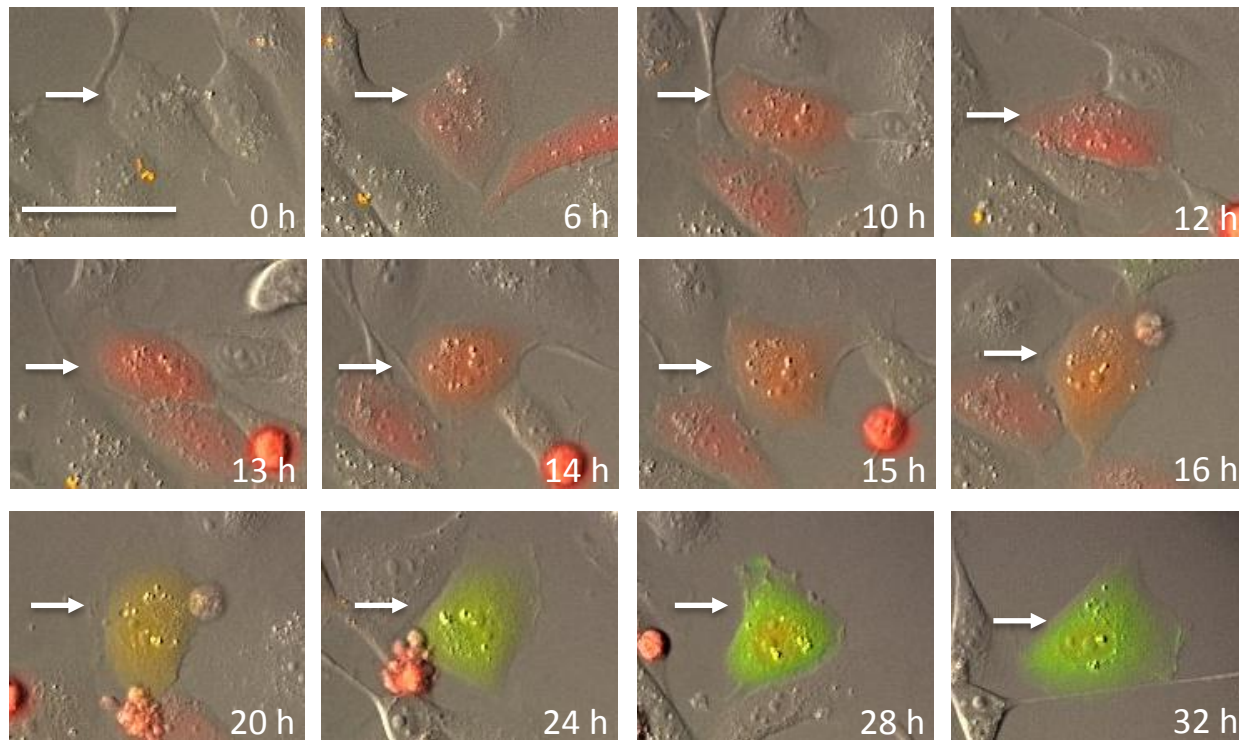

Maezawa et al. Supplemental Figure 1

Supplement: Supplementary file 1 — Fig. S1. Cell fluorescence over time. [file FEB4-6-16-s001.pdf]
